# Community paramedicine model of care: an observational, ethnographic case study

**DOI:** 10.1186/s12913-016-1282-0

**Published:** 2016-02-02

**Authors:** Peter O’Meara, Christine Stirling, Michel Ruest, Angela Martin

**Affiliations:** 1 La Trobe University, PO Box 199, Flora Hill, Victoria, 3552 Australia; 2 University of Tasmania, Private Bag 135, Hobart, Tasmania 7001 Australia; 3 County of Renfrew Paramedic Services, 9 International Drive, Pembroke, Ontario K8A 6W5 Canada; 4 La Trobe University, PO Box 199, Flora Hill, Victoria, 3550 Australia; 5 La Trobe Rural Health School, PO Box 199, Flora Hill, Victoria, 3552 Australia

**Keywords:** Community paramedicine, Rural health, Community engagement, Model of care, Integration, Leadership

## Abstract

**Background:**

Community paramedicine programs have emerged throughout North America and beyond in response to demographic changes and health system reform. Our aim was to identify and analyse how community paramedics create and maintain new role boundaries and identities in terms of flexibility and permeability and through this develop and frame a coherent community paramedicine model of care that distinguish the model from other innovations in paramedic service delivery.

**Methods:**

Using an observational ethnographic case study approach, we collected data through interviews, focus groups and field observations. We then applied a combination of thematic analysis techniques and boundary theory to develop a community paramedicine model of care.

**Results:**

A model of care that distinguishes community paramedicine from other paramedic service innovations emerged that follows the mnemonic **RESPIGHT**: **R**esponse to emergencies; **E**ngaging with communities; **S**ituated practice; **P**rimary health care; **I**ntegration with health, aged care and social services; **G**overnance and leadership; **H**igher education; **T**reatment and transport options.

**Conclusions:**

Community engagement and situated practice distinguish community paramedicine models of care from other paramedicine and out-of-hospital health care models. Successful community paramedicine programs are integrated with health, aged care and social services and benefit from strong governance and paramedic leadership.

## Background

There is a growing evidence base supporting the notion that community paramamedicine (CP) could form a new model of care that addresses some of the reform needs in the health sector [1]. The term CP covers emerging models of care that are a community-focused extension of the traditional emergency response and transportation paramedic model that has developed over the last 50 years. This new model of care calls on paramedics to apply their education, training and skills in “non-traditional” community-based environments and to embrace expanded scopes of practice [2, 3]. Models of care are theoretically informed and evidence based overarching designs for specific types of health care service [4]. Developing a CP model of care requires clarity about how CP services are delivered and how they differ from other related models such as extended care paramedics (ECP) that are well established in the United Kingdom [5] and the more recent mobile integrated healthcare (MIH) model found in the United States [6, 7].

A growing number of reports, evaluations and commentaries on CP programs are emerging, along with a more limited number of peer-reviewed empirical studies [8]. A small number of published studies have examined CP programs from a theoretical perspective [9], one of which utilised an observational ethnographic approach to develop a model built around the concepts of rural community engagement, emergency response, situated practice and primary healthcare. This is known as the RESP model [2].

The drive toward CP has come from a combination of health care service gaps in under-served communities and the growing professionalisation of the workforce, two common elements of new models of care. Firstly, there is a need to fill gaps in the delivery of healthcare services in rural or disadvantaged communities. This approach has been documented in Nova Scotia, Canada where a nurse/paramedic program was established in response to the lack of physicians in an isolated, island community [10]. Secondly, paramedics are increasingly accepted as healthcare providers who can make significant contributions toward improving the health and well-being of populations beyond traditional emergency response and transportation roles [11]. A manifestation of this growing professional recognition has been in England and Wales, where newly graduated paramedics are generally university educated and nationally registered health professionals [12]. These forces have enabled paramedics, physicians, health services and funding agencies the opportunity to loosen the bonds of the status quo and allowed paramedic roles to expand [13, 14]. These developments have the potential to challenge the professional domination over paramedicine that has been enacted through medical oversight [15, 16] as paramedicine evolves into an independent, autonomous health profession alongside nursing and allied health [17–21].

The emergence of more complex professional paramedic roles in the health sector, improved education, and the proliferation of CP programs raises questions about our understanding of what CP programs are and how they can be distinguished from ECP and MIH models [5, 6]. Understanding the intra-organizational and inter-professional relationships of paramedic services and paramedics with other health professionals and communities is a crucial step in efforts to understand these complex dynamics and the CP model of care [22].

High quality research has been undertaken in the U.K. that focuses on the introduction and evaluation of ECPs [23, 24]. A limitation of the relevance of this research is that, compared to community paramedics, ECPs operate in relatively reactive domains with limited levels of community engagement [25, 26]. Despite this, ECP research provides approaches to the monitoring and management of quality and safety that could equally apply to CP. The measurement of the more mosaic outcomes of CP programs, such as community engagement and integration with other health disciplines remains a major challenge to policy makers and researchers, with answers to these questions more likely to come from cross disciplinary research [27, 28].

Unlike ECP programs and to a lesser degree some of the CP programs, the MIH model is untested and appears to be highly reliant on high-level collaboration with often fragmented health systems. Publications on this model are largely opinion pieces that make a range of assumptions and unproven claims to advocate for its adoption [7], while others commenting on blogs and on-line media often confuse this model with CP. The MIH model is open to the criticism of being provider-centered, with patients treated as passive receivers of care instead of partners [14]. Another concern is that it entrenches medical dominance over paramedics which could potentially hold back the professional development of the paramedicine discipline [16].

While there have been a number of descriptive accounts of emerging paramedic roles in rural and remote settings [29–32], few have measured their impact on the health and well-being of communities [8, 10, 33]. Even fewer have applied a theoretical framework to the analysis of CP as an emerging model of care [2, 9].

## Methods

Building on a previous multiple case study in Australia [2], we used an observational ethnographic approach to describe and analyse a CP program in rural Canada. This enabled immersion in the case study setting and the generation of rich data, along with the opportunity to gather empirical insights into social practices that are normally “hidden” from the public view. To mitigate against the inherent threat of bias a wide range of participants were purposively recruited, a diversity of data collection methods were used (interviews, focus groups and direct observation of practice) and multiple researchers gathered and analysed these data [34].

### Setting

The field research was undertaken in Renfrew County, Ontario where a CP program has evolved in response to the changing healthcare needs of patients in specific communities and a diminution in the availability of essential healthcare services. This case study was selected purposively in keeping with the objective to develop theory because it was considered to be a particularly suitable case to illuminate and extend our understanding of the relationships and logic of CP programs.

Over time, this CP program has evolved into a coherent and sustainable program, initially consisting of four key components: Aging at Home Program; Paramedic Wellness Clinics; Ad hoc Home Visiting Program; and Community Paramedic Response Unit Program [35, 36]. A partnership model was used to frame the overall management of this CP program that enables effective collaboration with staff and other healthcare providers. At the time of the study there was no specific clinical governance model in place for the program, with paramedics working broadly within their existing scope of practice under the Ontario Provincial paramedic medical oversight model [37, 38].

### Data collection

Data collection took the form of direct observation of practice, informal discussions, interviews and focus groups undertaken by four researchers during the summers of 2012 and 2013. This multi-method approach captured the richness and diversity of the CP program within its natural setting and allowed issues to be studied in depth. It placed paramedic practice within the wider community context [39]. Participants were purposively recruited from the groups below to ensure rich, relevant and diverse data were obtained [40]:

- Community members, including patients, family and carers
- Paramedics and paramedic service managers from Renfrew County and the Greater Ottawa area
- Paramedicine educators in Ontario
- Physicians, nurse practitioners and other health care providers who interact with community paramedics
- Health economists and health service managers

Three focus groups of between 10 and 20 participants and 34 semi-structured interviews were conducted, allowing detailed, emotive responses, unconstrained by specific questions of a survey to points raised by the facilitator. Focus groups provided an opportunity to collate common issues or expectations and provide a stimulating and inclusive environment to encourage contributions.

We used the paramedic domains of practice identified in the Australian RESP Model - rural community engagement, emergency response, situated practice and primary health care - to locate the elements of this CP program and to develop the focus group and interview questions [2]. The focus group discussions and the expert informant interviews were recorded and transcribed, with each transcript coded and analysed inductively using classic thematic analysis techniques through manual methods [41]. This reflexive process approach, as opposed to the objectivist application of analysis procedures, enabled identification of common themes within the qualitative data, without the constraint of having to establish how these themes link together or explain all facets of the data [42].

Complementing the focus groups and interviews were field observations by two researchers (one an experienced health services researcher and the other a postgraduate research student) who independently accompanied community paramedics on calls and talked with other health professionals, patients, families and carers. This provided opportunities to observe the authenticity of community paramedic practice, engagement with the community and integration with the health system. Informal discussions with participants formed an important component of these observational phases of the data collection process and helped establish the general pattern of perception of the CP program [39]. The advantage of using this approach was that it shone a light on any discrepancies between rhetoric and reality. These sources of data facilitated a richer understanding of the behaviours and interactions of the community paramedic in a natural setting [43]. The resulting observations validated data from documents, the paramedic service, interviews and focus groups.

During the field observation phases the field researchers noted community paramedics’ practice during or immediately after events occurred, along with their own feelings and responses [44]. Content analysis of their field notes commenced during the data collection phases allowing categories to be developed, then tested against concepts and other data and then iteratively refined. The use of this range of data collection methods, a purposive sample of stakeholders as participants and multiple researchers enhanced the credibility, confirmability, dependability and transferability of the findings. These collectively demonstrate the trustworthiness of the findings [45].

### Data analysis

The domains of an Australian rural paramedic practice model (RESP Model) [2, 25, 31, 46–49] provided an initial conceptual framework for the analysis techniques with rural community engagement, emergency response, situated practice and primary healthcare forming initial categories for thematic analysis. Additionally boundary theory was used as a lens to identify and analyse how community paramedics create and maintain new role boundaries and identities in terms of flexibility and permeability [50].

Elements of boundary theory were applied to these data to explain the operation and dynamic of how this CP program operates. This approach has been previously applied in political science, anthropology, psychology and organizational studies to explain phenomena at either individual or organizational levels [50, 51]. Within health services research, it has been used to explore interprofessional practice in rural settings and paramedic models of service delivery [52, 53].

We looked at how the boundaries between individuals and the participating organisations influence how individuals perceive the professional identity of community paramedics and the local paramedic service, and how they negotiate the relationships between themselves and the CP program. Of particular interest were the key boundary characteristics of location and permeability [51]. Using boundary theory provided a means of considering the professional boundary interfaces of community paramedics, as well as exploring the physical, temporal and cognitive limits that define the CP model of care [51].

Those domains and factors associated with successful CP programs were brought together to describe a model of care. After analysis the following enabling factors were added to the four RESP model domains: integration with health, aged care and social services; governance and leadership; higher education; treatment and transport options.

### Ethical considerations

The La Trobe University Human Research Ethics Committee approved the research (Approval No. FHEC12/8) in compliance with the Helsinki Declaration. Ethical considerations included the importance of obtaining informed consent, and maintaining the anonymity and confidentiality of the participants. Informed written consent was obtained following the provision of a plain language information statement outlining the purposes of the research. Participation was voluntary and participants were able to withdraw at any time without giving any reason.

## Results

In this paper we address the challenge of analysing CP as an emerging model of care through a theoretical framework. We bring together the Australian RESP model [2] and new data from Canada to form a coherent framework (RESPIGHT) that describes the conceptual basis of a CP model of care, distinguish it from other innovations in paramedic service delivery and provides guidance to providers contemplating its introduction.

Our findings are presented in two parts: firstly, an overview of community paramedicine roles or domains of practice; and secondly, description of the enabling factors that have tangible or potential impacts on successful implementation and the continuing success of CP programs. In the discussion and conclusion the RESPIGHT community paramedicine model of care emerges from the synthesis of previous studies and these data from Ontario.

### Community paramedic domains

Data were initially aligned with the Australian RESP model domains of practice [2] and then modified to better reflect the observed situation in the field, in particular the potential to extend the reach of CP programs into urban environments. Apart from this modification, the earlier paramedic domains of practice were validated. The final domains adopted were: response to emergencies; engagement with community; situated practice; and primary health care.

#### Response to emergencies

In common with earlier studies, our participants expected paramedics to be available for emergency responses [54]. To some extent this creates a dilemma for paramedic services that seek to fulfil this central mission while offering community paramedic services during times of low emergency demand.

… providing emergency medical response is still there, we are not taking anything from that. This particular program [paramedic response unit] is actually adding more to that because they do have a first response capability and responsibility in these geographical areas. (P4)

Some days we don’t do any [CP] business at all, we just can’t, we’re on the road … it can be anything from transfers to emergency calls, so you might do two emergency calls but then you wind up doing three or four standbys and then a transfer … (P25)

The focus on emergency response roles illustrates how difficult paramedic services find it to expand beyond traditional activities or established professional domains when confronted with strong professional and community boundaries around the expected roles of paramedics. A culture of ‘stopping the clock’ has been well entrenched in paramedic services for the last 25 years and is only now coming to an end as the unintended consequences of focusing on expedited response and transport have been recognised [13, 55]. Observation of these relatively impermeable boundaries is consistent with an examination of how professional roles and boundaries are formed within primary health care teams [56]. The degree of permeability across and between domains determines which influences are allowed in or kept out of paramedic services. This influences the acceptability and success of new innovations such as CP programs that propose greater integration with health, aged care and social services [51, 57].

#### Engagement with community

Community or public engagement was rarely mentioned during the focus groups and interviews. However, participants demonstrated the value they placed upon community and professional engagement as they interacted with each other and when describing the skills they valued in each other. For instance, the paramedics working on the paramedic response unit highlighted the importance of good communication skills and the building of trusting relationships within the community.

This is a sub component of the whole umbrella and there are many facets of it … So we know them [clients of the adult day service] from multiple different angles. … we are so small a community that we know a lot of these people and we can watch them, help them to stay in their own home as long as possible, but then also transition them to the next step. (P15)

What are we offering? Basically a safety net, letting people know that there are people out there, the paramedics are there and we are trying to work with communities, CCAC [Community Care Access Centre], Mental Health, Alzheimer’s, whatever groups that we can work with, pulling together to work together for individuals in our community. Help them stay there longer and healthier. (P25)

Parker and colleagues [52] have discussed engagement within interprofessional practice; they examined the purpose of engagement and the level at which health professionals are willing and able to invest, ranging from direct care contexts and education of patients and staff to policy development and whole of community health service planning and provision. Reinforcing the importance of public engagement, Quick and Feldman give a nuanced description of public engagement that separates it into participation and inclusion [58]. Opportunities for public participation were demonstrated in the County of Renfrew through regular service planning meetings, shared activities between the paramedic service and other health and community agencies, involvement in discharge planning, and other ad hoc activities. The concept of inclusion is harder to demonstrate, except to say that almost all of the local study participants demonstrated ownership of the CP program and claimed some credit for its establishment.

#### Situated practice

Participants in one focus group and many in the interviews strongly identified with the situated practice domain. Their argument that some CP activities were only viable and sustainable if there was both a gap in current service delivery and a critical mass of patients is consistent with findings elsewhere [14, 59]. For instance, wellness clinics are of little value unless demand is consistently strong, otherwise home visiting was just as effective. Getting the program balance right was identified as an ongoing challenge and something that the participants found difficult to quantify.

The home visiting program is an excellent illustration of the value of situated practice with participants noting that there is much to observe in the home environment of patients, while observing that the power dynamic between the patient or client and the health professional is changed in this setting.

There’s often a big advantage to going into the home, because what you hear and see in a clinic setting and the discussion that happens in that setting is often very different from what you see and discuss in the home setting. … their discussion with you is very different from how another person might perceive the way that they are living, what their environment is, what the risks are in that situation. (P7)

… people in their own home have a higher sense of control and so sometimes they are more comfortable in having those discussions. It is more relaxed, they are sitting in their arm chair, they are not sitting in a sterile environment, so sometimes that discussion seems a bit more informal and some things might be shared that wouldn’t necessarily be in a more sterile environment. … [in the clinic] there can sometimes be a bit of a power dynamic between a physician and a client. (P7)

#### Primary health care

Doctors don’t do home visits anymore. Hardly ever! That’s why people call 911 all the time. We are short of physicians here and if you call and ask to make an appointment, it’s a minimum of three weeks. There’s our terminal problem. That’s why we’re training medics and nurses to cover night shifts in ‘Emerg’. (P24)

One of the important aspects of understanding the place of CP programs in primary health care is for community and health professionals to be more aware of the role capabilities and competencies of community paramedics. In this County, their scope of practice varies according to the availability of other health service providers and is strongly linked to the idea that community paramedics are working in an interprofessional practice setting as members of a collaborative team. This can be a difficult concept for paramedics themselves to come to grips with, while it is even more difficult for others to fully appreciate [60].

The establishment of closer ties between the paramedic service and other providers in this County highlights the challenges that hinder the development of a broader primary health care role for community paramedics. Professional boundaries between paramedics and other health professions are still being negotiated around different occupational histories, educational preparation and regulatory frameworks. One participant from an isolated community health service explained how their team, including physicians, a nurse practitioner and a social worker were grappling with the potential services that community paramedics could deliver in their area. While they could identify people who would benefit from these activities, they were still curious about what the paramedics could offer. Paramedic participants reported that other health professionals were surprised at the benefits of community paramedics visiting patients in their homes.

They’re amazed in ‘Emerg’ and Geriatric and in other clinics that we know this much about our patients … That there’s a little bit more history for them to work with. … We are the ones inside the house, we’re the ones who see the food, that see what’s in the fridge. (P25)

These ‘social work’ and health navigation role components of the CP program are consistent with what has been found elsewhere [48, 61]. Many of the participants suggested that patients and their families would benefit from being able to access a health professional to help them navigate the complexity of an often fragmented health system that can be characterised by a historical ‘disconnect’ between family medicine, acute care and community health services [52]. The high turn-over of staff in some community services was a particular frustration for clients and consumers who felt they were constantly explaining their situation and being re-assessed before services are provided.

… there’s a much easier relationship and a trust factor there between us and we can get things done a little bit faster sometimes. If we had the ability to tap into those resources and not have [consumer 11 and consumer 12] have to go through it then we would be able to provide that constant level of care and knowledge of the client that doesn’t always exist with the [existing system]. (P23)

### Enabling factors

We identified four factors associated with the successful implementation and future sustainability of CP programs. These newly described factors address the issue of whether CP models of care can be sustainable, given political will and adequate funding. These identified enabling factors are:

- Integration with health, aged care and social services
- Governance and leadership
- Higher education
- Treatment and transport options

#### Integration with health, aged care and social services

We’re connectors right? We don’t fix their problem with medical skills. We connect them to the resources they need so they don’t become a medical emergency. So it’s not that we train as ‘physios’ or occupational therapists or learn nursing skills, we don’t do any of that. We are not there to fix them; we’re there to connect them to the people that will. (P24)

Participants reported that the effective and deep integration of paramedic services with the local health system is a challenge when the provincial health system does not include the 52 municipally administered paramedic services even though they partially fund them.

Around twenty years ago there was downloading of paramedic services to the communities, so there was a complete divorce … paramedics belonged to a city, a region … the rest of healthcare belongs to the Ministry of Health. (FGB)

Nonetheless, the importance of effective integration between paramedic services and other providers was widely accepted amongst participants who recognised the evolution of patient-centered care in Canada and elsewhere [62].

If we are going to talk about patient-centered care then it is a team event and so we need to bring everybody in … from the physician all the way to the paramedic and everybody in-between. We all have to play together to make sure that this patient is dealt with in the most cost effective way, in the most appropriate way to get them in the hospital healthy enough to be discharged back home and make sure that that home support is there for them. Where does that home support come from? I think it comes from the paramedic. (P10)

#### Governance and leadership

While there is no published evidence that the separation of paramedic services from the health system has resulted in substantive changes in the quality or safety of clinical care [37], it nonetheless raises issues about the appropriateness of the current ‘medical oversight’ arrangements. This is particularly the case in CP programs that see a wide range of patients with chronic and complex conditions who do not present as emergencies. The certification and regulation of paramedic practice in North America is largely under the control of the medical profession through the process of ‘medical oversight’ or in some cases ‘medical direction’ [63]. The enactment of medical oversight in Ontario is provided through a unique and complex regional base hospital system that is focused on emergency medicine that arguably results in paramedic practitioners being relegated to the role of a ‘companion profession to medicine’ [37, 38]. This approach to the regulation of paramedics has been subject to some criticism in Ontario, with the Ontario Paramedic Association seeking the inclusion of paramedics into Ontario’s Health Professions Regulatory Advisory Council since 1995 [38, 64]. In this study a number of the physician participants in one of the focus groups suggested that this system might be inappropriate for CP programs providing primary healthcare, while several paramedic participants raised the issue of professional self-regulation in support of the Ontario Paramedic Association position.

In a number of countries, the oversight of paramedicine clinical standards is undertaken within clinical governance frameworks using the same principles as those used across a wide range of other health services. Physicians in these countries generally fulfil advisory roles within paramedic services, with limited executive authority [65]. In the U.K., paramedics are a registered health profession who come under the direct supervision of the Health Care and Professions Council that has oversight of entry-level education programs, keeps a register of paramedics, protects the title of paramedic and deals with practice issues through a disciplinary process [12]. The advantage of this type of regulation system is that it provides a viable pathway toward greater professional autonomy that makes individual paramedics more accountable for their practice and provides the means for the profession to take control of its own destiny [66].

Within the CP program context, moving toward an interprofessional practice and regulatory system has the advantage of giving paramedics the opportunity to develop greater levels of professional autonomy and accountability. Interprofessional practice “involves working together to achieve a common purpose of healthcare delivery, with mutual respect and improved health outcomes in contrast to different professions simply working side by side” [60].

#### Higher education

Participants identified the need for a broader education for paramedics, particularly in curricula related to health promotion and prevention [3, 35].

We came into this completely blind - completely cold. There was no training. We determined our own training needs. If you don’t know what you’re going to be doing exactly, how do you propose to determine what your training is going to be? (P24)

Educators identified the challenges of making acceptable changes to the current college education and training curricula that is filled to capacity with acute care topics and skills.

… there isn’t any education on health determinants, social determinants, the actual structure of how the system works … what kind of skills do we need to be able to possess or what skills do we need or what knowledge do we need to be able to successfully integrate, collaborate with our partners. (P3)

A lot of people who come into my program are looking for the lights and sirens and trauma and excitement, and when we start talking about something with a slower pace in community paramedicine, then we take them back. … a lot of students sort of walk in the door going, I want to drive fast, lights and sirens and car crashes and all that good stuff, and when you say, well in actual fact that’s about five percent of your career, 95 percent of your career looks much more like community paramedicine. (P10)

#### Treatment and transport options

One of the central motivations for establishing CP programs is to keep patients at home and avoid ‘inappropriate’ emergency department attendances or unnecessary hospital admissions [67]. For this vision to be enacted, pathways need to be established that are wider than the emergency medicine approach, with the social and cultural needs of isolated or disadvantaged patients addressed.

We’ve got to find a different place to take some of these people rather than into Emergency, find clinics, find alternatives and that’s what we are trying to do. To get them to a Day Hospital, Geriatric Hospital, something that they can have people come and work with them at their home. (P25)

In addition, there needs to be an acknowledgement of the difficulties that some patients and carers have navigating the health system.

[Paramedics] have to facilitate that link because often what happens is many of the patients, even if they are aware of the service, the actual physical act of making the connection is too complicated for them … even people in the system can’t navigate it … so you can imagine how intimidating it can be for someone who is rural, who is isolated, who doesn’t have a familiarity with the system. I think that facilitation for those linkages is really important. (P7)

## Discussion

### A community paramedicine model

The RESPIGHT CP model of care (Table 1) emerged from this Canadian case study and modification of the previously presented RESP model [2].

**Table 1:** RESPIGHT community paramedicine model of care

Domains of practice/enabling factors	Descriptions	Potential performance measures
**R**esponse to emergencies	Timely emergency responses remain the core business of paramedic services.	Monitor clinical outcomes. e.g. survival rates.
**E**ngaging with communities	Encouraging and embracing co-production with patient groups and/or communities.	Sustained participation in monitoring and management of programs. Evidence of inclusive community engagement.
**S**ituated practice	Key component of the model, giving it flexibility to respond to local needs and take account of existing resources.	Success in addressing the specific needs of communities. e.g. access, safety, equity, reliability.
**P**rimary health care	Expansion of practice from acute incidents to interprofessional care.	Monitor unnecessary ED presentations and hospital re-admissions. Records of preventative and health promotion activities.
**I**ntegration with health, aged care and social services	Both an enabler and a key benefit of the community paramedicine model.	Network analysis of communication and collaboration with key services.
**G**overnance and leadership	Paramedic leadership and effective interprofessional clinical governance systems.	Survey stakeholders and undertake clinical risk audits. Measure adverse events.
**H**igher education	Access to degree-level education for entry-level practitioners, consistent with other health professionals.	Map paramedicine program curricula against other health professions and community health needs.
**T**reatment and transport options	Development of clear and transparent clinical and social pathways for patients in collaboration with other health professionals, families and social services.	Cost-utility analysis comparing community paramedicine programs against established practice. Audit community paramedic referrals.

### Distinguishing features

The RESPIGHT model of care has been developed following observation and analysis of real world or ‘lived’ experience, it highlights the unique characteristics of CP that distinguish it from other related models of care such as mobile integrated healthcare in the U.S. and extended care paramedics in the U.K. These characteristics include the model’s adaptability to different settings, its use of inclusive engagement strategies to integrate with local communities and health systems, and the crucial role of paramedic leadership in the development of effective and appropriate governance structures and processes.

Community Paramedicine programs have emerged to fill service gaps in local health systems, with initiatives developed as unmet needs and opportunities presented. As a result, the development of CP programs have been organic and lacking a firm philosophical or theoretical framework. While the MIH programs share this weakness, they differ in other ways. They have been built on the application of positivism in pursuit of an all-encompassing health care model with limited input from the full range of stakeholders and it battles to address calls for patient-centered models of care [6, 7, 14, 62]. The experience of other qualitative researchers and CP pioneers supports the notion that the more reflexive iterative model development approaches that incorporate strong community engagement processes may be a better way to proceed when addressing the ‘messy problems’ that vary so much between communities [13, 29, 42, 68]. It is from this logic that the paramedic practice domain of ‘situated practice’ was first understood [2].

Additional features of the CP model identified in this study include the importance of strong integration and collaboration with health, aged care and social services; and the need to develop clinical and social pathways for ‘at risk’ population groups. These enabling factors are closely linked to the domain of engaging with local communities, whether through established institutions or using more inclusive strategies to connect with disenfranchised groups who are at risk of being excluded from access to health and community services [58, 69]. Paramedic services that implement the CP model of care have the capacity to be a broker for ‘at risk’ population groups who may only engage with the health system during times of crisis. Few other health professionals regularly make unscheduled contact with patients in their homes, workplaces or public places; this places community paramedics in an ideal position to observe and integrate the individual and social determinants of health into their practice. This ability to engage communities through a bottom-up, inclusive and community driven approach is a strength of the CP model of care.

### Additional enabling features

The public health philosophy of CP programs may play themselves out in the types of governance systems that emerge, with programs likely to embrace an interprofessional approach to clinical governance. There were hints of this in Renfrew County, despite the constraints of existing emergency medical service governance systems that mandate medical oversight from emergency physicians. When clinical governance was examined through the prism of boundary theory there was some ambiguity, with paramedics expressing only limited concern about ‘medical dominance’ in the current clinical leadership system. This lack of concern may have been a result of limited knowledge about alternate systems, such as the clinical governance models that operate in paramedic services outside of North America [66, 70, 71]. The CP model of care has the potential for paramedics to take more responsibility for their own professional practice issues and to develop higher levels of professional autonomy.

Any move toward a greater degree of paramedicine leadership is dependent and intertwined with higher levels of education [35] and appropriate legislative frameworks being established [21, 64]. This profesional transition could see the manisfestation of challenges to established professional boundaries as the position of paramedics as ‘sub-professional’ and subservient health providers changes to a situation where paramedics take lead roles in partnership with other health professionals [38, 66, 72]. Such a system could see a separation emerge between governance, management and clinical delivery levels, rather than being part of one role in the form of a medical director [73].

This study has several strengths and limitations. The case study was conducted in one Canadian county using ethnographic data from one CP program with four key components. The data collection included a broad array of participants and encompassed the cultural setting of the program. The findings are not therefore representative of all CP programs but are generalizable on the basis of theory. The study scope did not encompass client outcomes and future studies need to address this. The study used the previously developed domains of practice from the Australian RESP model as a conceptual framework to guide the data collection. While some criticise using a priori concepts, it importantly builds on existing knowledge, as demonstrated by the expansion of the original Australian model to encompass Canadian CP work.

## Conclusions

Through this study, we have provided a picture of the working environment of community paramedics and how their roles and that of paramedic services may develop in the future. We have analysed the current and future place of CP programs in the health system of the future through the development of a CP model of care. When combined with earlier studies, the findings indicate that successful CP programs are integrated with local health systems, have viable treatment and referral options for sub-acute and chronic patients, are built on broad paramedic education and have inclusive governance systems. These characteristics distinguish community paramedicine models of care from other paramedicine and out-of-hospital models. The RESPIGHT community paramedicine model of care can be used as the basis for informed dialogue, debate and discussion about community paramedic roles and how this innovation can contribute toward improvements in health outcomes through stronger integration with the health system [74].
